# Clinical study on the use of advanced magnetic resonance imaging in lupus nephritis

**DOI:** 10.1186/s12880-022-00928-w

**Published:** 2022-11-30

**Authors:** Yan-Xia Chen, Wa Zhou, Yin-Quan Ye, Lei Zeng, Xian-Feng Wu, Ben Ke, Hao Peng, Xiang-Dong Fang

**Affiliations:** 1 Department of Nephrology, The Second Affiliated Hospital of Nanchang University, No. 1 Minde Road, Nanchang, 330006 China; 2 Department of Nephrology, Jiangxi Provincial People’s Hospital, Nanchang, 330006 China; 3 Image Center, The Second Affiliated Hospital of Nanchang University, Nanchang, 330006 China

**Keywords:** Lupus nephritis, Advanced magnetic resonance imaging, Histopathology of kidney, C3, C1q

## Abstract

**Objectives:**

To investigate the correlation between the histopathology of the kidney and clinical indicators in patients with lupus nephritis (LN) using magnetic resonance imaging (MRI).

**Methods:**

A total 50 female participants were enrolled in the study. Thirty patients with LN were divided into types 2, 3, 4, and 5, according to their pathological features. The control group consisted of 20 healthy female volunteers. Serum creatinine, C3, C1q, and anti-ds-DNA were measured. Conventional MRI, DTI, DWI, and BOLD scanning was performed to obtain the FA, ADC, and R2* values for the kidney.

**Results:**

Compared with the control group, FA and the ADC were decreased in patients with LN, while the R2* value was increased (*P* < 0.05). The overall comparison of the SLEDAI (Activity index of systemic lupus erythematosus) score, total pathological score, AI, and serum creatinine C3 showed that these were significantly different between the two groups (*P* < 0.05). FA and the ADC were negatively correlated with urinary, blood ds-DNA, and serum creatinine and positively correlated with C1q (*P* < 0.05). The R2* value was positively correlated with urinary NGAL, blood ds-DNA, and serum creatinine (*P* < 0.05). FA and the ADC were negatively correlated with the SLEDAI score, total pathological score, AI, CI, nephridial tissue C3, and C1q. The R2* value was positively correlated with the SLEDAI score, total pathological score, AI, CI, nephridial tissue C3, and C1q (*P* < 0.05).

**Conclusions:**

MRI examination in female patients with LN was correlated with pathologic test results, which may have clinical significance in determining the disease’s severity, treatment, and outcome.

## Introduction

Systemic lupus erythematosus (SLE) is an autoimmune disease that involves multiple bodily systems. Based on the histopathology of the kidney, patients with SLE are often diagnosed with lupus nephritis (LN), and this condition is one of the leading causes of death in patients with SLE [1]. Renal biopsy pathology is the gold standard used to confirm the severity and activity of renal lesions in patients with LN. However, renal biopsy is invasive, with a high risk of bleeding, and the biopsy specimens are not comprehensive, as the repeated punctures required to define the disease’s pathological changes and treatment outcomes cannot be executed in a short time. Accordingly, there is an urgent need to develop an alternative method to renal biopsy, to guide diagnosis and observe treatment and prognosis. Recently, the study of magnetic resonance imaging (MRI) technology in kidney disease has attracted much attention. Advanced MRI techniques such as DTI (diffusion tensor imaging), DWI (diffusion weighted imaging), and BOLD (Blood oxygen level dependent) can reflect the pathophysiological changes of kidney disease from multiple aspects. The principle of DWI and DTI (an advanced form of DWI) is to calculate the apparent diffusion coefficient (ADC) by quantifying the signal value of water molecules based on their Brownian motion [2]. Renal BOLD imaging can be used to evaluate renal oxygen metabolism [3]. In this study, fractional anisotropy (FA), ADC and the apparent spin–spin relaxation rate (R2*) of LN patients were obtained by advance MRI, and the correlation between the renal FA, ADC, R2* and pathological and laboratory activity indicators was investigated, in an attempt to find an early noninvasive diagnosis and evaluation technique for LN.

## Materials and methods

### Subjects

The LN group consisted of 30 female patients with LN and nephrology admitted to our hospital between January 2016 and December 2019. Inclusion criteria were (1) conformation to the diagnostic criteria of SLE, as revised by American College of Rheumatology in 1997, to obtain a SLEDAI score, and (2) diagnosis via renal biopsy pathology. The control group consisted of 20 healthy female volunteers who underwent examination. Inclusion criteria were (1) no history of diabetes mellitus, hypertension, and kidney disease; (2) no current kidney disease; (3) had not recently received any drug with obvious nephrotoxicity; and (4) acceptable kidney function according to various laboratory test indices. Before examination, all patients received no medicine for 8 h and fasted for at least 6 h. Water was withheld for 4 h before the examination [4]. All patients performed exercise bouts with end-expiratory breath holding before the MRI test. This study was conducted in accordance with the declaration of Helsinki and approved by the Ethics Committee of our hospital. All patients who participated in the study signed an informed consent form.

### Advanced MRI test

Conventional MRI and DTI, DWI, and BOLD scanning was performed on the kidney using the GE Singa HDxt GE3.0 T MR system. Post-image processing techniques were used to obtain the FA, ADC, and R2* values for the kidney.

### Relevant laboratory test indices

All patients with LN received 99mTc-DTPA (99mTc pentanoic acid) renal dynamic imaging in the Department of Nuclear Medicine of the hospital 1 week before and after the MRI test to obtain the SKGFR (the single kidney glomerular filtration rate). Levels of mALB and urinary NGAL were obtained from urine samples. Serum creatinine, C3, C1q, and anti-ds-DNA were obtained from blood samples.

### Renal pathological examination

Subjects underwent renal biopsy under the guidance of color ultrasonography 7 days before and after the MRI test. Hematoxylin–eosin staining, periodic acid-schiff stain, Masson staining, and periodic acid-GMS were used for kidney tissues. For immunopathology, a fast, sensitive, direct immunization was utilized. The fluorescence intensity distribution of complement C3 and C1q was expressed with “ ± , + , +  + , +  +  + ” and replaced by “0.5, 1, 2, 3” when processed statistically. Based on ISN/RPS (International Society of Nephrology/Society of renal pathology) (2003) criteria, patients with LN were classified as follows: type II (n = 4), type III (n = 6, including type III merged with type V), type IV (n = 12, including type IV merged with type V), and type V (n = 8). The groups did not differ by GFR (glomerular filtration rate) or age. Using the semi-quantitative scoring method of the NIH (National Institutes of health), the AI (Activity index) and CI (Chronic index) were calculated for the patient group.

### Collection of advanced MRI data

#### MRI scanning method

Singa HDxt 3.0 T magnetic resonance scanner (General Electric, Wisconsin, WI, USA) is used. After routine T1WI and T2WI renal scanning, renal DWI imaging was performed. DWI adopts single shot echo plane imaging (SS-EPI) sequence, TR 6316 ms, TE 63.1 ms, matrix 96*180, FOV 340 mm*240 mm, NEX 8 times, b value is 800 s/mm2. The scanning center layer is located at the center layer of the kidney, with 6 layers. The surface phased array coil is used. The breath gating technology is used to eliminate the artifacts caused by the movement of abdominal organs, and the fat suppression technology is used to suppress the abdominal fat signal. Subjects received conventional double kidney T1W1 and T2W1 scanning using BOLD imaging.

#### DWI and DTI using a single-shot echo-planar sequence

The scanning center level was in the center of the kidney. Six levels with a layer thickness of 8 mm were used. Using DWI images showing good corticomedullary demarcations, the central zone of renal hilum was selected to draw the regions-of-interest (ROI). ROI was drawn at the clearly demarcated level of renal skin and medulla, and the cortical contour was delineated with lines to avoid renal cysts and chemical shift artifacts as much as possible. Use a circular tool to draw three circular areas of about 15-25mm2 in the upper, middle and lower polar circles of the kidney. Average values were assigned for the ADC (apparent diffusion coefficient) and FA (fractional anisotropy) of the corresponding renal medulla.

BOLD imaging used the Functool 9.4.05a software package of ADW4.4 Workstation's Functool Workstation (General Electric, Wisconsin, WI, USA) for post-processing of the scanning data. The R2* post-process platform was selected. The software system automatically generated the R2* image. The R2* value was obtained using the same method as above.

The images were measured and analyzed jointly by two MRI attending physicians, and they were discussed when they had different opinions.

### Statistical methods

Data analysis was performed using SPSS Statistics 22.0. Measurement data conforming to the normal distribution were presented as “ ± s”. Comparisons between multiple groups were performed using ANOVA. The LSD (Least Significant Difference)—t test was used for pairwise comparisons, if there was the homogeneity of variance, while the Tamhane T2 test was used if there was the heterogeneity of variance. Data correlation was analyzed by Pearson correlation analysis. A *p* value < 0.05 was considered to indicate a statistically significant difference.

## Results

### Comparison of advanced MRI in all groups

Compared with the control group, FA and the ADC were significantly different in patients with LN (*p* < 0.05 for all types) and were decreased significantly in the type IV group. However, the R2* value was increased, and this difference reached statistical significance in the type IV and V groups (all *p* < 0.05) (Table 1).

**Table 1:** Comparison of fMRI in each group ($\overline{x}$ ± s)

	Cortex FA um^2^/ms	Medulla FA um^2^/ms	Cortex ADC 10^−3^ mm^2^/s	Medulla ADC 10^−3^ mm^2^/s	Cortex R2* xHz	Medulla R2* xHz
Control group	0.31 ± 0.02	0.44 ± 0.03	2.29 ± 0.1	2.20 ± 0.09	15.87 ± 0.70	32.20 ± 1.26
Type II	0.27 ± 0.01*	0.37 ± 0.02*	2.13 ± 0.89*	2.12 ± 0.09	16.49 ± 0.39	33.39 ± 0.74
Type III	0.26 ± 0.02*	0.37 ± 0.03*	2.06 ± 0.09*	2.04 ± 0.87*	16.82 ± 0.90	34.18 ± 1.16*
Type IV	0.24 ± 0.01*#※	0.32 ± 0.02*#※	1.95 ± 0.14*#※	1.90 ± 0.13*#※	19.95 ± 1.78*#※	37.21 ± 2.09*#※
Type V	0.26 ± 0.02*◎	0.36 ± 0.03*◎	2.05 ± 0.11*	2.02 ± 0.13*◎	17.62 ± 1.47*◎	34.40 ± 1.21*◎
F value	24.328	44.915	20.221	14.731	22.933	22.141
*P* value	< 0.001	< 0.001	< 0.001	< 0.001	< 0.001	< 0.001

Compared with normal control group, *P < 0.05; compared with type II, #*P* < 0.05; compared with type III, ※*P* < 0.05; compared with type IV, ◎*P* < 0.05

### Comparison of laboratory test indices in patients with LN

The overall comparison of mALB and urinary NGAL showed statistically significant differences between the groups (*p* < 0.05 for all groups), of which the mALB was the highest. Furthermore, urinary NGAL was increased significantly in the type IV group. However, the levels of creatinine, C3, C1q, and anti-ds-DNA showed no differences between the groups (*p* > 0.05 for all groups) (Table 2).

**Table 2:** Comparison of laboratory indexes in LN groups ($\overline{x}$ ± s)

	UMA mg/l	Urinary NGAL ug/l	SCR umol/l	Blood C3 g/l	Blood C1q mg/l	Blood ds-DNA IU/ml
Type II	143.00 ± 90.89	38.49 ± 10.85	55.00 ± 12.52	1.00 ± 0.14	216.83 ± 37.09	60.61 ± 23.35
Type III	927.33 ± 375.96	68.89 ± 19.38	78.17 ± 17.51	0.94 ± 0.24	198.88 ± 40.32	92.51 ± 33.55
Type IV	2704.75 ± 694.95#	118.15 ± 48.55#※	98.02 ± 15.40	0.94 ± 0.35	176.65 ± 31.00	122.32 ± 19.00#
Type V	3408.63 ± 760.49#※	78.61 ± 36.96	74.75 ± 25.31	0.86 ± 0.24	213.81 ± 51.21	103.18 ± 42.52
F value	3.570	4.244	1.491	0.259	1.854	1.581
*P* value	0.028	0.014	0.240	0.854	0.162	0.240

*UMA* urine microalbumin, *NGAL* Neutrophil gelatinase associated lipocalin, *SCR* serum creatinine; Compared with type II, #*P* < 0.05; compared with type III, ※*P* < 0.05; compared with type IV, ◎*P* < 0.05

### Comparison of SLEDAI scores and histopathological indices of LN in all groups

Comparison of the SLEDAI score, total pathological score, AI, and serum creatinine C3 showed there were statistically significant differences between the groups (*p *< 0.05), with the biggest differences being seen for the SLEDAI score, total pathological score, AI, and serum creatinine C3 in the type IV group. CI and C1q deposits in renal tissues did not differ between the groups (*p* > 0.05 for all groups) (Table 3).

**Table 3:** Comparison of SLEDAI score and pathological indexes in LN groups ($\overline{x}$ ± s)

	SLEDAI score	Total pathological score	AI	CI	Nephridial tissue C3	Nephridial tissue C1q
Type II	4.25 ± 2.63	4.25 ± 1.5	2.75 ± 0.96	1.50 ± 0.58	0.88 ± 0.75	1.00 ± 0.71
Type III	6.17 ± 2.40	5.00 ± 1.67	3.33 ± 1.03	1.67 ± 0.82	1.33 ± 0.82	1.50 ± 0.84
Type IV	8.33 ± 2.23#	8.50 ± 1.68#※	6.08 ± 1.38#※	2.33 ± 0.89	2.25 ± 0.87#※	2.00 ± 0.85#
Type V	7.13 ± 1.73#	4.88 ± 1.55◎	3.13 ± 0.99◎	1.88 ± 0.64	1.75 ± 0.89	1.75 ± 0.71
F value	3.868	12.786	15.564	1.688	3.280	1.733
*P* value	0.021	< 0.001	< 0.001	0.194	0.037	0.185

*LN* lupus nephritis, Compared with type II, #*P* < 0.05; compared with type III, ※*P* < 0.05; compared with type IV, ◎*P* < 0.05

### Correlation of advanced MRI and laboratory test indices in patients with LN

Pearson correlation analysis showed that the FA, ADC, and R* values were significantly negatively correlated with urinary NGAL, blood ds-DNA, and serum creatinine (*p* < 0.05 for all comparisons), and significantly positively correlated with C1q (*p *< 0.05 for all comparisons). There was no correlation between the FA or R2* value and mALB or between FA and blood C3 (*p* > 0.05 for all comparisons) (Table 4).

**Table 4:** Correlation between fMRI and laboratory parameters in LN group

fMRI	*UMA R(P)*	Urinary NGAL *R(P)*	*SCR R(P)*	Blood C3 *R(P)*	Blood C1q *R(P)*	Blood ds-DNA *R(P)*
Cortex FA	− 0.214(< 0.001)	− 0.731(< 0.001)	− 0.620(< 0.001)	0.356(0.054)	0.607(< 0.001)	− 0.618(< 0.001)
Medulla FA	− 0.191(0.312)	− 0.635(< 0.001)	− 0.500(0.005)	0.307(0.099)	0.530(0.003)	− 0.496(0.005)
Cortex ADC	− 0.401(0.028)	− 0.745(< 0.001)	− 0.616(< 0.001)	0.582(0.001)	0.488(0.006)	− 0.739(< 0.001)
Medulla ADC	− 0.451(0.012)	− 0.761(< 0.001)	− 0.598(< 0.001)	0.531(0.003)	0.508(0.004)	− 0.734(< 0.001)
Cortex R2*	0.375(0.041)	0.853(< 0.001)	0.661(< 0.001)	− 0.482(0.007)	− − 0.576(0.001)	0.725(< 0.001)
Medulla R2*	0.339(0.067)	0.840(< 0.001)	0.673(< 0.001)	− 0.527 0.003	− 0.589(0.001)	0.768(< 0.001)

*LN* lupus nephritis, *UMA* urine microalbumin, *NGAL* Neutrophil gelatinase associated lipocalin, *SCR* serum creatinine

### Correlation of advanced MRI and SLEDAI scores and histopathological indices in patients with LN

FA and the ADC were significantly negatively correlated with the SLEDAI score, total pathological score, AI, CI, and nephridial tissue C3 andC1q. The R2*value was significantly positively correlated with the SLEDAI score, total pathological score, AI, CI, and nephridial tissue C3 and C1q (*p* < 0.05 for all comparisons) (Table 5).

**Table 5:** Correlation between fMRI and SLEDAI score and pathological indexes in LN group

fMRI	SLEDAI score *r(P)*	*Total pathological score r(P)*	AI *r(P)*	CI *r(P)*	Nephridial tissue C3 *r(P)*	Nephridial tissue C1q *r(P)*
Cortex FA	− 0.686(< 0.001)	− 0.898(< 0.001)	− 0.802(< 0.001)	− 0.776(< 0.001)	− 0.632(< 0.001)	− 0.556(0.001)
Medulla FA	− 0.564(0.001)	− 0.773(< 0.001)	− 0.703(< 0.001)	− 0.64(< 0.001)	− 0.579(< 0.001)	− 0.451(0.012)
Cortex ADC	− 0.723(< 0.001)	− 0.814(< 0.001)	− 0.715(< 0.001)	− 0.795(< 0.001)	− 0.676(< 0.001)	− 0.599(< 0.001)
Medulla ADC	− 0.735(< 0.001)	− 0.856(< 0.001)	− 0.779(< 0.001)	− 0.778(< 0.001)	− 0.687(< 0.001)	− 0.582(0.001)
Cortex R2*	0.770(< 0.001)	0.926(< 0.001)	0.866(< 0.001)	0.733(< 0.001)	0.685(< 0.001)	0.675(< 0.001)
Medulla R2*	0.754(< 0.001)	0.951(< 0.001)	0.864(< 0.001)	0.697(< 0.001)	0.715(< 0.001)	0.698(< 0.001)

*LN* lupus nephritis

## Discussion

*fMRI has high safety for being performed without using paramagnetic contrast material* A previous study showed that in patients with CKD (chronic kidney disease), advanced MRI including DTI, DWI and BOLD imaging was important in the evaluation of renal histopathology and kidney function change [5]. The kidney is rich in blood flow and the motion of water molecules is active and complicated. The ultrastructure of the kidney can be determined by testing the diffusion of the water molecules. The ADC reflects the diffusion of water molecules in the renal cortex and medulla. This has diagnostic value for evaluating chronic kidney disease as the ADC and FA value decrease when kidney disease occurs [6–8]. Our study showed the ADC and FA for both the cortex and medulla declined in the group with LN, compared with the control group, with the largest decline in the type IV group. In Class III and IV proliferative disease, complexed that deposit in the subendothelium injure endothelial cells. These deposits had access to the vascular space and may activate circulating myeloid cells that express FC receptors, allowing them to infiltrate the renal tissue. By contrast, subepithelial deposits, found in Class V disease, injure podocytes but elicit a less severe inflammatory response, as they contacted only the urinary space [9]. Pairwise comparisons of the type II, III, and IV groups showed significant differences, which also conform to the most obvious characteristic of type IV LN activity. Consistent with a previous study, the ADC value in the cortex was higher than that in the medulla [10]. The FA value in the cortex was smaller than that in the medulla, for that medulla is a small radial tubular structure, with many glomeruli, and while the anisotropic diffusion of water molecules is apparent, the FA rate is reduced [11]. Because of more blood flow in the medulla rather than the cortex, the medulla of the kidney is extremely sensitive to ischemic responses and is prone to hypobaric injury [12, 13]. Our study showed that the R2* value in patients with LN was increased, compared with the control group. The increase in the type IV group was the most significant. Furthermore, the medullary R2* value was higher than that for the renal cortex, indicating that hypoxic injury had occurred. R2* is proportional to the amount of deoxygenated hemoglobin in the tissue, the ratio of oxygenated hemoglobin to deoxygenated hemoglobin is related to the partial pressure of oxygen in the blood. In addition, the partial pressure of oxygen in the capillary is in equilibrium with the partial pressure of oxygen in the surrounding tissue, so the signal changes detected by BOLD MRI can be interpreted as changes in the partial pressure of oxygen in the tissue. The increase of R2* indicates that the proportion of local tissue deoxyhemoglobin increases and the partial pressure of tissue oxygen decreases. On the contrary, the decrease of R2* indicates the decrease of deoxygenated hemoglobin concentration and the increase of local oxygen partial pressure [14–16]. Simon-Zoula et al. [14] reported that there is a certain correlation between the R2* value of cortex and medulla of healthy volunteers and age. The R2* value of renal cortex and medulla increases with age, indicating that the medulla was more hypoxic in the elderly than in the young. One study of BOLD imaging in diabetic nephropathy has shown decreased R2* values in the renal medulla, suggesting increased renal oxygenation, which is considered to be related to decreased medulla oxygen consumption in diabetic nephropathy [17]. Xiao et al. founded that the R2* ratio of renal medulla/cortex is a sensitive indicator to reflect the normal function of kidney. Compared with the healthy control group, the R2* ratio of renal medulla/cortex in the kidney with acute graft rejection is significantly lower [18].

The comprehensive evaluation of LN activity is important for medication administration and outcome observation in clinical practice. The SLEDAI score is currently the most commonly used standard for evaluating SLE disease activity with the help of other indicators [19]. When the GFR of patients with LN is disrupted, albumin diafiltration increases significantly. Urinary albumin increases when it cannot be reabsorbed by the nephric tubule, indicating early kidney injury. Therefore, mALB has an important role in the diagnosis of early kidney injury in SLE. NGAL is widely found in normal human body tissues. Urinary NGAL increases after kidney injury [20], and recent studies [21, 22] have shown that it is a biomarker for acute kidney injury, which increases significantly in patients with active LN and is positively correlated with the SLEDAI. The complement system plays an important role in multiple kidney diseases, including LN [23]. Our study has demonstrated that a reduction of C3 is related to kidney disease in patients with LN. C1q is the initial factor in classical pathway activation, which is involved in the development of LN. The anti-ds DNA antibody, unique to systemic lupus erythematosus, is deposited in the kidney and results in further injury. Our study showed that the SLEDAI score, mALB and urinary NGAL were statistically different across the groups of patients. However, there was no difference in C3, C1q, and ds-DNA. The reason for such results may be related to our selection of patients. Using an immunologic test in the kidney tissues of patients with LN, Gaya et al. [24] observed that large C3 and C1q deposits were detected in the kidney. Further examination in our study found that the total pathological score, AI, and nephridial tissue C3 were significantly different across the patient groups, but the CI and nephridial tissue C1q did not differ.

Pearson’s correlation analysis showed a strong correlation between advanced MRI and renal pathology. Thus, examination of the kidney by advanced MRI in patients with LN appears to be useful in the early recognition of the severity, treatment and prognosis of LN, particularly for patients who refuse renal biopsy or for whom it is contraindicated.

This study establishes advanced MRI intervals in control subjects and patients with LN and identifies the correlation between renal advanced MRI values and pathological and laboratory activity indicators. However, this study has certain limitations. First, all patients included in the study were female, and male patients with SLE might present with more severe LN. The effect of gender on the activity of SLE and renal pathology has not yet been determined. In addition, none of the enrolled patients had serious complications such as neuropsychiatric or hematological system damage, and the effect of renal appearance was not defined. Therefore, further studies are required to define the clinical utility and broader indications of advanced MRI.

## Conclusion

There was correlation between MRI examination and pathologic test results in female patients with LN, indication clinical significance of advanced MRI including DTI, DWI and BOLD imaging in determining the disease’s severity, treatment, and outcome.

## Data Availability

Data related to the current study are available from the corresponding author on reasonable request.

## Abbreviations

LN: Lupus nephritis
MRI: Magnetic resonance imaging
SLE: Systemic lupus erythematosus
fMRI: Functional MRI
DTI: Diffusion tensor imaging
DWI: Diffusion weighted imaging
BOLD: Blood oxygen level dependent
FA: Fractional anisotropy
ADC: Apparent diffusion coefficient
R2*: Relaxation rate
99mTc-DTPA: 99MTc pentanoic acid
ISN/RPS: International Society of Nephrology/Society of renal pathology
GFR: Glomerular filtration rate
NIH: National Institutes of health
AI: Activity index
CI: Chronic index
LSD: Least significant difference
